# Soluble Co‐Inhibitory Immune Checkpoint Molecules Are Increased in Patients With Polymyalgia Rheumatica Without Significant Correlations With Clinical Status: A Case‐Control Study

**DOI:** 10.1002/acr2.70045

**Published:** 2025-05-09

**Authors:** Elvis Hysa, Dario Camellino, Christian Dejaco, Matteo Bauckneht, Giampaola Pesce, Silvia Morbelli, Marcello Bagnasco, Maurizio Cutolo, Eric L. Matteson, Marco A. Cimmino, Daniele Saverino

**Affiliations:** ^1^ Laboratory of Experimental Rheumatology and Academic Division of Clinical Rheumatology, Department of Internal Medicine and Department of Experimental Medicine (DIMES), University of Genova Genova Italy; ^2^ DIMES, University of Genova, Division of Rheumatology, “La Colletta” Hospital, Local Health Trust 3 Genova Italy; ^3^ Department of Rheumatology and Immunology, Medical University of Graz, Graz, Austria, and Department of Rheumatology, Hospital of Brunico (SABES‐ASDAA), Department of Rheumatology, Teaching Hospital of the Paracelsus Medical University Brunico Italy; ^4^ Nuclear Medicine Unit, IRCCS Ospedale Policlinico San Martino, Department of Health Sciences, University of Genova Genova Italy; ^5^ Department of Internal Medicine, Immunology Unit, University of Genova, Diagnostic Laboratory of Autoimmunology, IRCSS Ospedale Policlinico San Martino Genova Italy; ^6^ Nuclear Medicine Unit, Department of Medical Sciences University of Turin Turin Italy; ^7^ Department of Internal Medicine and Medical Specialties University of Genova Genova Italy; ^8^ Laboratory of Experimental Rheumatology and Academic Division of Clinical Rheumatology, Department of Internal Medicine University of Genova, IRCSS Ospedale Policlinico San Martino Genova Italy; ^9^ Mayo Clinic College of Medicine and Science Rochester Minnesota; ^10^ Laboratory of Experimental Rheumatology and Academic Division of Clinical Rheumatology, Department of Internal Medicine University of Genova Genova Italy; ^11^ DIMES, University of Genova, Diagnostic Laboratory of Autoimmunology IRCSS Ospedale Policlinico San Martino Genova Italy

## Abstract

**Objective:**

A dysregulated immune response is involved in the pathogenesis of polymyalgia rheumatica (PMR) and giant cell arteritis (GCA). These diseases have been reported as immune‐related adverse events in patients with cancer treated with immune checkpoints inhibitors. In this cross‐sectional study, the relationship between soluble immune checkpoint molecules (sICMs) and clinical/imaging features of PMR and GCA was investigated.

**Methods:**

Consecutive patients with PMR diagnosed according to the criteria by Bird et al were compared with age‐ and sex‐matched healthy controls. Patients with PMR and overlapping GCA had to also satisfy the 1990 ACR classification criteria for GCA. All patients underwent standardized clinical, laboratory examination, and ^18^F‐fluorodeoxyglucose positron emission tomography/computed tomography scans. The sICM anticytotoxic T Ly‐4, the programmed cell death protein 1 (PD‐1), and PD‐1 ligands PD‐L1 and PD‐L2 were measured by enzyme‐linked immunosorbent assay.

**Results:**

Forty patients (80% women, mean age 76 years, and mean disease duration 88 days) were assessed. Of these, 30 had isolated PMR and 10 had PMR with GCA. Patients showed significantly higher concentrations of all sICMs compared with controls (*P* < 0.001). Conditional logistic regression revealed the strong discriminative capacity of these molecules between patients and healthy controls, with PD‐1 showing complete separation among groups (effect size = 0.78) and PD‐L1 (odds ratio [OR] 134.33, *P* < 0.001) and PD‐L2 (OR 63.00, *P* < 0.001) demonstrating the strongest ability to distinguish patients from controls. Correlations between sICM levels and clinical features were generally weak or absent, with no significant differences based on disease phenotype or glucocorticoid exposure. Results were similar in glucocorticoid‐naive patients.

**Conclusion:**

sICMs are significantly elevated in PMR and GCA and strongly differentiate patients from healthy controls. Although they do not correlate with clinical or imaging features, their consistent elevation in active disease might suggest a complex interplay between innate and adaptive immunity.

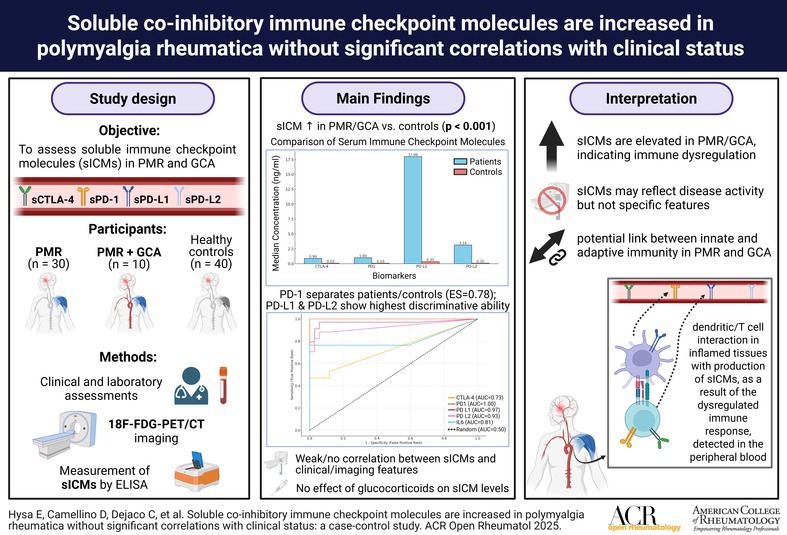

## Graphical abstract

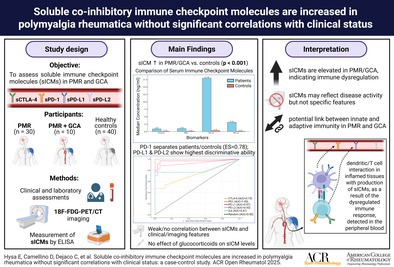

## INTRODUCTION

Polymyalgia rheumatica (PMR) and giant cell arteritis (GCA) are closely associated inflammatory diseases of older adults.^1^ PMR is characterized by pain and stiffness in the neck, shoulder, and pelvic girdles, often accompanied by systemic inflammatory symptoms, such as fever and weight loss, and increased acute phase reactants.^2^ GCA is the most common large vessel vasculitis (LVV) in patients aged ≥50 years and is characterized by a granulomatous inflammation of the aorta and its branches.^3^ PMR features are seen in up to 40% to 60% of newly diagnosed GCA, and approximately 16% to 21% of patients with PMR develop GCA at follow‐up.^4^, ^5^, ^6^ Accordingly, the concept of GCA‐PMR spectrum disease (GPSD) has been consolidated by experts in literature.^7^, ^8^, ^9^

The pathogenetic mechanisms underlying these two conditions remain partially elusive. In GCA, histologic evidence points to infiltrates of effector T helper (Th) 1/17 cells and macrophages actively secreting interleukin (IL) 6, IL‐17, and interferon (IFN) γ.^10^ Conversely, the main cellular elements involved in PMR appear to be macrophages, with histologic findings showing scarce infiltrates of T lymphocytes and B cells.^11^, ^12^

Recent data suggest an altered immune cell inhibitory signaling in GCA pathophysiology with a critical role of the cytotoxic T lymphocyte‐associated protein 4 (CTLA‐4) pathway.^13^ This is not demonstrated in patients with isolated PMR.

Physiologically, immune checkpoint molecules (ICMs) are receptors that inhibit immune cell activation. They comprise the CTLA‐4 and the programmed cell death protein 1 (PD‐1).^14^ CTLA‐4 blocks T cell activation by competing with CD28 for binding with CD80/86, resulting in the inhibition of T cell activation.^14^ A soluble form of CTLA‐4 (sCTLA‐4) can also bind to its ligands and exert immunomodulatory effects via competitive binding.^15^

Similarly, PD‐1 is crucial for ending immune responses by binding its ligands, PD‐L1 and PD‐L2, which are mainly expressed on antigen‐presenting and other immune cells.^16^ Soluble forms of PD‐1 (sPD‐1) and PD‐L1 (sPD‐L1) have also been described, with sPD‐1 displaying a potential immunostimulatory effect by blocking the connection with PD‐L1, thus impeding the inhibitory message transduction.^17^

Increased serum sCTLA‐4 concentrations have been reported in several autoimmune diseases,^15^, ^18^ and a reduced PD‐1/PD‐L1 signaling has been detected in the synovial microenvironment of patients with rheumatoid arthritis (RA), leading to the recent development of drugs stimulating this endogenous immune‐inhibitory pathway.^19^, ^20^

The rationale for studying the role of soluble ICMs (sICMs) in PMR and GCA is provided by the accumulating evidence of drug‐induced PMR and/or GCA in patients with cancer treated with immune checkpoint inhibitors (ICIs).^21^, ^22^, ^23^

## PATIENTS AND METHODS

Consecutive patients diagnosed with PMR according to the criteria of Bird et al^24^ were prospectively enrolled. Patients with PMR and overlapping GCA had to also satisfy the 1990 American College of Rheumatology criteria for GCA.^25^ Patients treated with glucocorticoids (GCs) for >15 days were excluded. A group of age‐ and sex‐matched healthy controls was also included. Patients and controls signed an informed consent (CONSAZHQA_0001). This study was performed with the authorization of the Ethical Committee of San Martino Polyclinic Hospital, Genoa, Italy (EC 2013/4301).

### Clinical and laboratory assessment

All patients underwent a complete clinical history and subsequent standardized physical examination. Disease duration, morning stiffness, presence of fever, weight loss, headache, jaw claudication, visual disturbance, spontaneous pain in the girdles and in the spine, limb claudication, and previous GC therapy were recorded.

White blood cells, platelets, hemoglobin, C‐reactive protein (CRP), and erythrocyte sedimentation rate (ESR) values were retrieved from patients’ routine laboratory evaluations performed in the week preceding the ^18^F‐fluorodeoxyglucose positron emission tomography/computed tomography (^18^F‐FDG PET/CT) scan. Blood samples were collected the same day as the PET scan, before the infusion of FDG. Serum concentrations of sCTLA‐4, sPD‐1, sPD‐L1, and sPD‐L2 were measured by enzyme‐linked immunosorbent assay (EMELCA Bioscience, Clinge, the Netherlands) according to the manufacturer's instructions (see Supplementary Table 1 for further details). Each sample was diluted 1:10 and tested in duplicate. Serum IL‐6 was measured by enzyme‐linked immunosorbent assay (ImmunoTools GmbH, Friesoythe, Germany) according to the manufacturer's instructions. Each sample for IL‐6 concentrations was diluted 1:10 and tested in duplicate (Supplementary Table 1).

### PET/CT acquisition

All patients underwent FDG PET/CT scanning. After a minimum of 6 hours of fasting, a dose of 4.8 to 5.2 MBq of ^18^F‐FDG per kilogram body weight was injected through a peripheral vein catheter. Patients were placed in a quiet room and instructed to remain still. Data acquisition started ≥60 minutes after intravenous administration of ^18^F‐FDG. Patients underwent simultaneous FDG PET and CT imaging from the skull base to the thighs using an integrated PET/CT scanner (Hirez; Siemens Medical Solutions, Knoxville, TN, USA). In some patients, the scan also included legs and feet if they appeared to be involved clinically. PET scan raw data were reconstructed by means of ordered subset expectation maximization, and attenuation correction was performed using the CT scan raw data. The entire CT dataset was fused with the 3‐dimensional PET scan images using an integrated software interface (Syngo Image Fusion; Siemens, Erlangen, Germany) to create anatomic images superimposed with the FDG uptake.

### Image analysis

Joint and vascular uptake were scored both semiquantitatively with a visual score and quantitatively by an experienced nuclear medicine physician (M Bauckneht). All scans were reviewed by a second nuclear medicine physician (SM) to confirm protocol adherence and quality control. Regions of interest (ROIs) were placed on the anatomic CT scan images to identify four aortic segments (ascending, arch, descending, and abdominal), subclavian arteries, common carotid arteries, and iliac and femoral arteries. ROIs were drawn on the theoretical vessel wall to exclude the uptake of the blood inside the vessel lumen. A further region was drawn within the left ventricular chamber using the PET scan image to estimate the tracer concentration in the arterial blood (blood pool [BP]). To assess joint metabolism, CT‐based ROIs were bilaterally drawn on the glenohumeral, sternoclavicular, and coxofemoral joints and on the trochanteric and ischiatic bursae.

Arterial FDG uptake was quantified by calculating the mean standardized uptake value (SUV) within each ROI. To account for the contribution of FDG activity in the blood, results were expressed as the ratio between the mean SUV value of each ROI and BP ROI (SUV/BP), expressing true arterial wall metabolic activity; joint FDG uptake was considered without the BP ratio.

Arterial and joint uptake was visually graded using a four‐point scale as proposed by Walter et al^26^: 0 = no uptake present, 1 = uptake present but lower than liver uptake, 2 = similar to liver uptake, and 3 = uptake higher than liver uptake. To determine the prevalence of each finding, these scores were further subdivided as “negative” (0 and 1) and “positive” (2 and 3). In accordance with recently published consensus recommendations for PET scan interpretation in the context of LVV and PMR imaging, patients demonstrating grade 2 findings were deemed as possibly indicative of LVV, whereas those with grade 3 positive results were characterized as exhibiting active LVV, with this conclusion supported by evidence level II, grade B.^27^ For each patient, the sum of the four‐point score of vascular and joint uptake was recorded as total vascular score, with a maximum score of 36, and total joint score (TJS), also considering the uptake in cervical and lumbar interspinous bursae, with a maximum score of 36.

### Statistical analysis

Analysis was performed using IBM SPSS Statistics software. The comparison of means was evaluated with Student's *t*‐test and analysis of variance with Bonferroni correction. Medians were compared with the Mann‐Whitney or Kruskall‐Wallis tests. The correlations between variables were evaluated with the Pearson test if normally distributed and with the Spearman rho test if nonparametric.

To account for the matched case‐control design, conditional logistic regression was applied to assess the association between sICMs and PMR/GCA, using two different cutoff strategies: the median and 75th percentile of control values. In case of complete separation between cases and controls, alternative metrics, including standardized effect size (Cohen d) and mean differences, were used to quantify the magnitude of association. Receiver operating characteristic (ROC) curve analysis was performed to determine the discriminative ability of sICMs in differentiating patients with PMR/GCA from healthy controls. Optimal cutoffs were identified using the Youden index, along with their corresponding sensitivity and specificity values. Statistical significance was assumed as *P* < 0.05.

## RESULTS

### Patient characteristics

Forty patients were included: 30 presented with clinically isolated PMR and 10 with GCA and associated PMR. Six (15%) patients were taking GCs, with a median dosage of 18.75 mg/day (range 7.5–25) and a median duration of treatment of 10 days (range 3–15). Ten patients (25%) exhibited grade 3 LVV at PET/CT. Of these, 4 presented as cranial GCA with overlapping PMR symptoms, whereas the remaining 6 had PMR with LV‐GCA. Other patient characteristics are outlined in Table 1.

**Table 1:** Demographic and clinical characteristics of patients*

Characteristics	Data
Total number	40
Male, n (%)	8 (20)
Median age (range), years	76 (50–85)
Patients with isolated PMR, n (%)	30 (75)
Patients with PMR + GCA, n (%)	10 (25)
Already taking a GC, n (%)	6 (15)
Systemic manifestations, n (%)	17 (42.5)
Peripheral arthritis, n (%)	10 (25)
Median disease duration (range), days	87.5 (4–1,086)
Median MS (range), minutes	60 (0–360)
Mean ± SD WBC, 10^9^/L	8.4 ± 1.6
Mean ± SD PLT, 10^9^/L	318.8 ± 92.4
Mean ± SD Hb, g/L	124 ± 14
Mean ± SD ESR, mm/h	67.7 ± 33.7
Median CRP, mg/L	31.6 (range 2–124.3)
TJS, mean ± SD	18 ± 6
TVS, mean ± SD	15 ± 10
Presence of grade‐3 LVV, n (%)	10 (25)

* CRP, C‐reactive protein; ESR, erythrocyte sedimentation rate; GC, glucocorticoid; GCA, giant cell arteritis; Hb, hemoglobin; LVV, large vessel vasculitis; MS, morning stiffness; PLT, platelet; PMR, polymyalgia rheumatica; TJS, total joint score; TVS, total vascular score; WBC: white blood cell.

### Comparison between patients and controls

**Figure 1:**
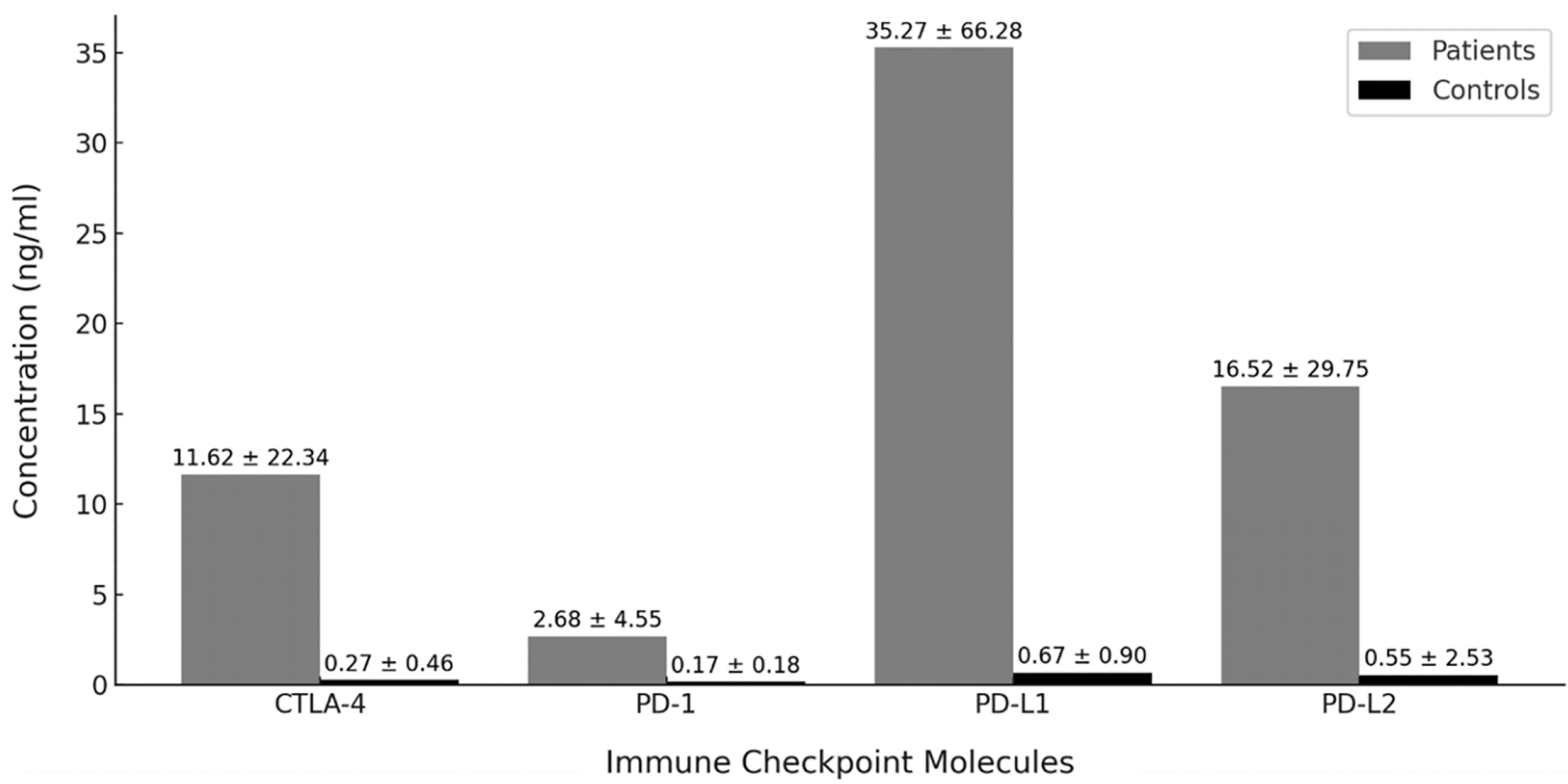
Comparison between the mean and SDs between serum concentrations of the soluble immune checkpoint molecules in patients (gray) versus controls (black). CTLA‐4, cytotoxic T lymphocyte‐associated protein 4; PD‐1, programmed cell death protein 1; PD‐L, programmed cell death protein ligand.

The mean ± SD serum levels of sCTLA‐4, sPD‐1, sPD‐L1, and sPD‐L2 were significantly higher in patients than in healthy controls (*P* < 0.001 for all comparisons; Figure 1). The mean ± SD IL‐6 concentration was also significantly higher in patients than in controls (114.3 ± 140.7 vs 3.1 ± 1.88 pg/mL, *P* < 0.01).

### Correlations and subgroup analyses

Male patients had a higher mean concentration of sCTLA‐4 than female patients (12.7 vs 0.1 ng/mL, *P* = 0.03), whereas concentrations of PD‐1, PD‐L1, and PD‐L2 were comparable. Serum CTLA‐4 concentrations directly correlated with disease duration (r = 0.34, *P* = 0.03), whereas plasmatic PD‐L1 levels were inversely associated with the duration of morning stiffness (r = −0.34, *P* = 0.04). No other correlations were observed between the remaining sICMs with the clinical, laboratory, and imaging characteristics (Table 2).

**Table 2:** Correlations between soluble immune checkpoint molecules and IL‐6 with clinical, laboratory, and imaging features*

Table of correlations	CTLA‐4		PD‐1		PD‐L1		PD‐L2		IL‐6	
	Rho	*P*	Rho	*P*	Rho	*P*	Rho	*P*	Rho	*P*
Age	0.06	0.71	0.11	0.51	0.20	0.21	0.27	0.09	0.07	0.67
TVS	0.08	0.62	0.14	0.38	0.1	0.54	0.09	0.59	0.02	0.9
TJS	0.07	0.68	0.21	0.19	0.28	0.08	0.21	0.20	0.02	0.91
Disease duration	**0.34**	**0.03**	0.01	0.95	−0.02	0.87	0.03	0.86	0.09	0.57
MS	0.09	0.58	0.27	0.09	**−0.34**	**0.04**	−0.14	0.41	−0.1	0.54
CRP	−0.19	0.25	−0.09	0.58	−0.05	0.78	0.08	0.61	−0.02	0.86
ESR	0.11	0.49	−0.10	0.53	−0.09	0.56	0.13	0.43	−0.10	0.53
PLT	0.03	0.85	−0.16	0.32	−0.05	0.76	0.11	0.49	0.14	0.40
Hb	−0.14	0.39	−0.15	0.37	0.21	0.20	0.02	0.91	−0.21	0.19
WBC	0.08	0.62	−0.14	0.39	0.21	0.21	0.16	0.32	−0.12	0.48

* Significant *p*‐values with the related rho coefficients are reported in bold. CRP, C‐reactive protein; CTLA‐4, cytotoxic T lymphocyte‐associated protein 4; ESR, erythrocyte sedimentation rate; Hb, hemoglobin; IL‐6, interleukin 6; MS, morning stiffness; PD‐1, programmed cell death protein 1; PD‐L, programmed cell death protein ligand; PLT, platelet; TJS, total joint score; TVS, total vascular score; WBC, white blood cell.

Concentrations of IL‐6 did not correlate with those of sICMs, nor with the clinical and imaging parameters. The differences in the concentration of sICMs between patients affected by both PMR and GCA compared with isolated PMR were not statistically significant, with *P* values ranging between 0.12 and 0.65 depending on the analyzed sICM (see Supplementary Table 2). This was also the case when patients were stratified according to the presence of systemic manifestations (ie, fever and/or loss of weight), ischemic symptoms (ie, headache, visual disturbances, and jaw claudication), and peripheral arthritis and/or tenosynovitis (Supplementary Tables 3–7). The six patients already taking GC treatment had sICM values comparable to those of the 34 GC‐naive patients. (Supplementary Table 8, see the paragraph below “Data related to GC‐naive patients”). Patients with grade 3 LVV did not significantly differ from the others in terms of mean concentrations of sICMs and IL‐6 (Supplementary Table 9).

### Results of the conditional logistic regression analysis

At conditional logistic regression analysis, all molecules showed significant discriminative capacity between patients and controls (*P* < 0.05). PD‐L1 demonstrated the strongest discriminative ability using the 75th percentile cutoff (odds ratio [OR] 134.33, *P* < 0.0001), followed by PD‐L2 (OR 63.00, *P* < 0.0001). For PD‐1, complete separation occurred between cases and controls (no overlapping values between groups), indicating excellent discriminative power that precluded OR calculation. CTLA‐4 showed moderate discriminative ability (OR 8.56, *P* = 0.0001), whereas IL‐6 showed significant but comparatively lower discrimination (OR 10.33 at the 75th percentile, *P* = 0.0105). These results suggest that elevated concentrations of sICMs, particularly PD‐1, PD‐L1, and PD‐L2, strongly distinguish patients with PMR/GCA from controls. For PD‐1, we used standardized effect size to quantify the magnitude of difference between groups. The standardized effect size was 0.78 (with 0.2, 0.5, and 0.8 conventionally representing small, medium, and large effects, respectively), confirming a substantial difference between cases and controls (Tables 3 and 4).

**Table 3:** Conditional logistic regression analysis of soluble immune checkpoint molecules in patients with polymyalgia rheumatica and giant cell arteritis compared with age‐ and sex‐matched controls*

Molecule	Median cutoff	OR (median)	75th cutoff	OR (75th)	*P* values
CTLA‐4	0.10	8.56	0.10	8.56	0.0001
PD‐1	0.10	NA	0.10	NA	<0.0001
PD‐L1	0.35	39.00	0.90	134.33	<0.0001
PD‐L2	0.10	63.00	0.10	63.00	<0.0001
IL‐6	3.40	3.44	4.73	10.33	0.01

* CTLA‐4, cytotoxic T lymphocyte‐associated protein 4; IL‐6, interleukin 6; NA, not applicable; OR (median), odds ratio using the control group median as the cutoff; OR (75th): odds ratio using the control group 75th percentile as the cutoff; PD‐1, programmed cell death protein 1; PD‐L, programmed cell death protein ligand.

**Table 4:** Programmed cell death protein 1 alternative analysis

Metric	Value	Interpretation
Mean difference	2.52	Average elevation in cases vs controls
Effect size	0.78	Large standardized difference
Distribution	Nonoverlapping	Complete separation between groups

### ROC curve analysis, diagnostic performance, and sensitivity analyses

**Figure 2:**
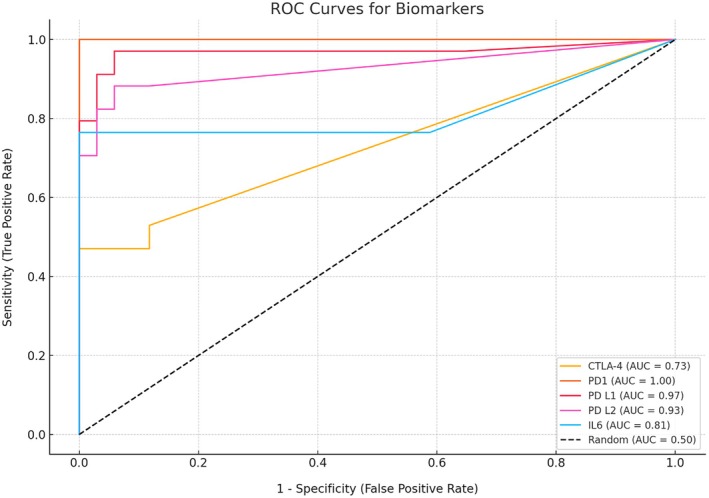
ROC curves of soluble immune checkpoint molecules in polymyalgia rheumatica/giant cell arteritis: discriminative capacity between patients with polymyalgia rheumatica/giant cell arteritis and healthy controls. AUC, area under the curve; CTLA‐4, cytotoxic T lymphocyte‐associated protein 4; IL‐6, interleukin 6; PD‐1, programmed cell death protein 1; PD‐L, programmed cell death protein ligand; ROC, receiver operating characteristic.

At ROC curves, PD‐1 showed the highest diagnostic accuracy (area under the curve [AUC] 1.000, optimal cutoff 0.96 ng/mL, sensitivity 100%, and specificity 97.5%) followed by PD‐L1 (AUC 0.971, optimal cutoff 1.98 ng/mL, sensitivity 97.5%, and specificity 92.5%) and PD‐L2 (AUC 0.919, optimal cutoff 0.51 ng/mL, sensitivity 90.0%, and specificity 92.5%). CTLA‐4 and IL‐6 showed good discrimination with AUCs of 0.744 and 0.814, respectively (Figure 2).

Sensitivity analyses were performed to assess potential confounding. Stratification by sex showed consistent discriminatory performance of sICMs in both men (PD‐1 AUC 1.000, PD‐L1 AUC 1.000, and PD‐L2 AUC 0.930) and women (PD‐1 AUC 1.000, PD‐L1 AUC 0.963, and PD‐L2 AUC 0.919). Age‐stratified analysis (<70 vs ≥70 years) revealed comparable AUCs for PD‐1 (1.000 vs 0.999), PD‐L1 (0.983 vs 0.965), and PD‐L2 (1.000 vs 0.889). CTLA‐4 and IL‐6 maintained moderate‐to‐good discrimination across all subgroups (AUC range 0.700–0.930 and 0.702–0.854, respectively). These results suggest that the association between sICMs and PMR/GCA is robust across age and sex subgroups (Supplementary Table 10).

### Data related to GC‐naive patients

#### Comparison with healthy controls in GC‐naive patients

The serum concentrations of the sICMs were significantly higher also in the subgroups of GC‐naive patients (n = 34) compared with healthy controls (Supplementary Figure 1; all *P* values <0.001). IL‐6 serum levels were also significantly higher in this subgroup compared with those of healthy controls (77 pg/mL vs 3.2 pg/mL, *P* < 0.001).

#### Correlations and subgroup analyses

A weak‐to‐moderate positive correlation emerged between serum PD‐L1 and TJS (r = 0.38, *P* = 0.03; Supplementary Table 11). No significant differences were found in the median sICM concentrations between PMR/GCA versus isolated PMR (Supplementary Table 12) or when stratifying by vascular/musculoskeletal manifestations (Supplementary Tables 13–17), although CTLA‐4 showed a trend toward higher values in patients with hand tenosynovitis (median 11.8 [range 4.2–15.5] vs median 0.1 [range 0.1–10.2], *P* = 0.07).

#### Conditional logistic regression analysis and ROC analyses in GC‐naive patients

Conditional logistic regression confirmed the strong discriminative capacity of sICMs between patients and controls, particularly for PD‐L1 (OR 132.47, *P* < 0.0001) and PD‐L2 (OR 60.00, *P* < 0.0001). PD‐1 showed complete separation between groups (effect size 0.80). ROC analysis demonstrated excellent discrimination for PD‐1 (AUC 1.000), PD‐L1 (AUC 0.969), and PD‐L2 (AUC 0.927), whereas CTLA‐4 and IL‐6 showed good‐to‐moderate performance (AUCs of 0.730 and 0.813, respectively). The results were consistent with those of the total cohort analysis (Supplementary Table 18).

## DISCUSSION

ICIs are increasingly used for treating several types of cancer.^28^ Despite being successful weapons in the treatment of malignancies, they are associated with a multitude of immune‐mediated adverse effects, including endocrine, gastroenterologic, renal, cutaneous, and articular manifestations.^29^ New‐onset inflammatory arthritis occurs in approximately 5% to 7% of patients with cancer treated with ICIs.^30^, ^31^ Other possible manifestations include myositis, sicca syndrome, and sarcoidosis, as well as PMR and GCA.^32^

The occurrence of PMR and GCA in patients treated with ICIs also hints at an imbalance of these regulatory pathways in the pathogenesis of their idiopathic forms. In fact, temporal artery specimens from patients with active GCA have shown low levels of PD‐L1 and high levels of PD‐1 transcripts, suggesting defective immunoregulatory mechanisms that lead to vascular inflammation in GCA.^33^ In contrast, arteries from healthy donors showed high levels of PD‐L1 transcripts but almost a complete absence of PD‐1 transcripts, indicating a lack of T cells in the normal vessel wall.^34^ Additionally, the use of an anti–PD‐1 antibody in a mouse model of GCA increased vascular inflammation and T cell infiltration.^33^, ^34^

**Figure 3:**
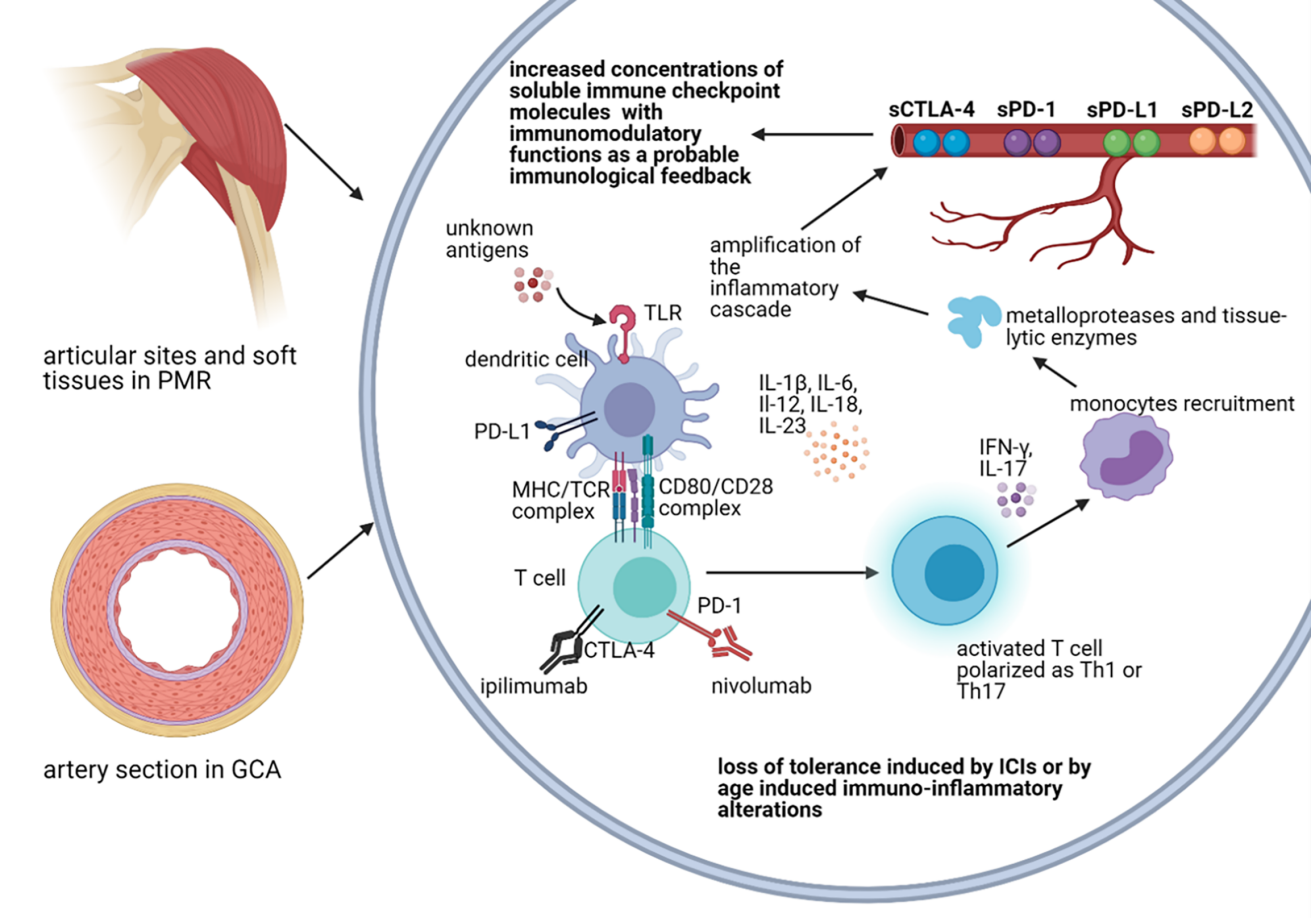
Possible role of soluble immune checkpoint molecules in PMR and GCA. CTLA‐4, cytotoxic T lymphocyte‐associated protein 4; GCA, giant cell arteritis; ICI: immune checkpoints inhibitor; IL, interleukin; INF, interferon; MHC, major histocompatibility complex; PD‐1, programmed cell death protein 1; PD‐L, programmed cell death protein ligand; PMR, polymyalgia rheumatica; s, soluble; TCR, T cell receptor; Th, T helper.

In a cross‐sectional study on patients with GCA, circulating PD‐1+ Th cells were reduced in comparison those of healthy controls.^35^ Circulating Th cells expressing the negative checkpoint V‐domain Ig‐containing suppressor of T cell activation (VISTA) were also reduced in number. On the other hand, arteritic lesions from diagnostic temporal artery biopsies were increased in VISTA‐expressing cells and in PD‐L1–expressing cells.^35^ These findings suggest a deficiency of the immune checkpoint activity in GCA, a hypothesis that is supported by cases of ICI‐induced vasculitides.^36^ Although the expression of the PD‐1/PD‐L1 immune checkpoint has not been reported yet in patients with isolated PMR, there are several cases of onset of PMR symptoms in patients treated with anti–PD‐1/anti–PD‐L1 agents, again suggesting a potential role of PD‐1/PD‐L1 signal in PMR pathogenesis^37^ (Figure 3).

In the current study, we analyzed the soluble forms of CTLA‐4 and PD‐1 and its ligands in patients with PMR and GCA and assessed whether different clinical, laboratory, and imaging characteristics could be attributed to the different expression and functions of immune checkpoint markers. Patients in this study had significantly higher levels of sCTLA‐4, sPD‐1, sPD‐L1, and sPD‐L2 than healthy controls. This finding is in line with the existing literature in several other autoimmune diseases.^38^ However, we did not find significant correlations between concentrations of these markers and disease duration, morning stiffness, CRP, ESR, or the total burden of vascular and articular inflammation as assessed by FDG PET/CT scan. Indeed, the isolated significant associations between sCTLA‐4 and disease duration and between PD‐L1 and morning stiffness duration were weak and likely not relevant from a clinical and pathophysiologic point of view. Although certain differences, such as the trend toward lower CTLA‐4 serum concentrations in patients with PMR with concomitant GCA compared with isolated PMR, may be biologically plausible given the immunoregulatory role of CTLA‐4, their clinical relevance remains uncertain. One possible explanation is that in GCA, the heightened immune activation and altered regulatory T cells function may lead to increased CTLA‐4 use at the tissue level, reducing its circulating levels as a compensatory response. Supporting this, recent evidence suggests that while regulatory T cells in GCA are numerically reduced and functionally impaired they up‐regulate CTLA‐4 and are more susceptible to depletion, indicating a potential increase in local demand for CTLA‐4–mediated immunoregulation.^13^ However, without established minimal clinically important differences for sICMs in autoimmune diseases, the significance of these variations remains unclear and warrants further investigation.^18^

In GC‐naive patients, a positive correlation emerged between PD‐L1 and the TJS. This finding is intriguing given that PD‐L1 expression has been found to be elevated in the synovial tissue of patients with inflammatory arthritis, where it may represent an attempted compensatory mechanism to control local inflammation.^39^ Similar mechanisms have been described in RA, where increased PD‐L1 expression in the synovium correlates with disease activity and joint involvement. However, the modest strength of this correlation in our cohort, combined with its absence in the total population, suggests that its clinical relevance requires validation in larger studies.

We also tested whether there are differences in circulating ICMs in patients with associated GCA. No differences were detected in comparison with patients with isolated PMR, either in terms of clinical presentation (eg, presence or absence of headache, jaw claudication, or systemic inflammation) or imaging‐detected vasculitis. Moreover, no significant differences were observed in the concentrations of sICM between GC‐treated patients and those naive to prednisone. However, it is important to note that this subgroup of GC‐treated patients may have been limited by the small sample size of GC‐treated patients (n = 6).

Preliminary data from a pharmacovigilance study suggested preferential involvement of the CTLA‐4 pathway in the development of ICI‐induced GCA, as patients with temporal arteritis were more likely to have received anti–CTLA‐4 treatment.^40^ Furthermore, a recent multicenter clinical study of patients with ICI‐induced GCA revealed that individuals receiving dual therapy with CTLA‐4 and PD‐1/L1 blockers more frequently exhibited cranial involvement, including permanent visual loss, compared with those treated with PD‐1 blockers alone, highlighting the significant role of CTLA‐4 signaling.^41^ In contrast, a study by Zhang et al pointed to a significant role for the PD‐1 pathway.^33^ In this respect, a recent systematic review summarizing data from 44 patients with ICI‐induced GCA revealed that most individuals developing GCA after ICI treatment were receiving PD‐1/PD‐L1 blockers (57%).^23^ A trial of abatacept, a CTLA‐4‐Ig used for the treatment of RA, in patients with GCA showed a slightly significant increase in relapse‐free survival at 12 months compared with controls.^42^ Although the overall results of this trial were positive, the small effect of abatacept may suggest that this pathway is not a pivotal target in GCA, and indeed, an industry‐sponsored trial on the use of subcutaneous abatacept in patients with GCA (NCT03192969) has been withdrawn. The results of a phase III trial on the use of abatacept monotherapy in patients with early‐onset PMR, without associated GC for the first 12 weeks of treatment, showed that the effect of abatacept alone is not strong enough to justify larger studies in early PMR (primary endpoint of low disease activity reached in 50% of patients receiving abatacept vs 22% of individuals of the placebo group, *P* = 0.07).^43^

An interesting question to consider is whether ICI‐induced rheumatic diseases serve as reliable models for studying their idiopathic disease counterparts. A report on six cases of ICI‐induced PMR showed imaging characteristics comparable with those of usual PMR.^44^ In a mixed cohort of patients with ICI‐induced PMR and cases from a literature review, many showed atypical features: peripheral synovitis (including unusual sites such as the elbow), positivity of autoantibodies, and sicca syndrome.^22^ In a cohort of 14 patients with ICI‐induced PMR, peripheral arthritis was present in 57%, compared with 28% of the control group composed of 43 patients with “classic” PMR.^45^ In our study, 25% of patients presented with peripheral arthritis, but its presence was not correlated with the concentrations of sICM.

The study of ICI‐induced rheumatic disease is in its infancy. Several mechanisms have been postulated to explain the pathophysiology of immune‐related adverse events, including a pre‐existing and latent autoimmunity.^46^ Another point suggested by the ICIs is that PMR, considered by some authors an autoinflammatory disease rather than an autoimmune one,^12^ may be triggered also by autoimmune mechanisms.^12^ At the same time, it is plausible that primary PMR and ICI‐induced PMR could represent distinct conditions, with the former potentially primarily driven by innate immunity (macrophages) and the latter by the aberrant activation of T cells resulting from ICI treatment. In fact, a recent cross‐sectional, observational study from the Netherlands compared the clinical, laboratory, and imaging characteristics of patients with ICI‐induced PMR with those of primary PMR, suggesting that ICI‐induced PMR may have a milder course with less inflammation as assessed by ^18^F‐FDG PET/CT.^47^ Moreover, a recent systematic review identified differences in sex prevalence, with ICI‐induced PMR showing a higher occurrence in men, and lower incidence of remitting seronegative symmetric synovitis with pitting edema, greater prevalence of normal or slightly elevated markers of inflammation, and lower relapse rate compared with primary PMR.^23^

The results of our study demonstrate that ICMs are involved in PMR and GCA, as highlighted by the increase of their soluble forms in this cohort. However, the CTLA‐4 and PD‐1 pathways do not appear to play a central role in GPSD. This is supported by the absence of correlation with articular and vascular manifestations in our patients and by the limited efficacy of abatacept in treating GCA and PMR, which contrasts with its success in other autoimmune rheumatic diseases characterized by predominant T cell activation, such as RA.^48^, ^49^ The elevated serum concentrations of sCTLA‐4 observed in our cohort indicate that patients with PMR may inherently trigger an immune‐regulatory mechanism to curb T cell hyperactivity. However, this response alone might be insufficient, as indicated by the limited efficacy of abatacept (CTLA‐4‐Ig fusion protein) in PMR,^43^ which suggests that the innate immune system could also play a predominant role in the disease process^12^ (Figure 3).

Although our study demonstrates significantly higher levels of sICMs in patients compared with controls, the interpretation of differences between patient subgroups (eg, PMR vs PMR + GCA or GC‐treated vs GC‐naive) requires caution. The absence of established minimal clinically important differences for sICMs in rheumatic diseases, combined with our sample size, makes it challenging to determine the clinical relevance of these subgroup variations. For instance, the trend toward lower CTLA‐4 levels in patients with PMR with concomitant GCA compared with those with PMR alone might reflect biologic differences in immune regulation, but larger studies are needed to establish clinically meaningful thresholds and confirm these findings.

The strengths of the study include the prospective inclusion of consecutive, unselected patients and the accurate clinical, laboratory, and imaging assessment of all the patients. To the best of our knowledge, this is the largest study analyzing serum ICMs in patients with PMR and GCA.

The limitations of this study include the fact that a few patients were already taking GC treatment at the time of the assessments. However, treatment duration was short, cumulative dosage was low, and these patients had mean concentrations of sICMs comparable with GC‐naive patients. Although we did not systematically collect data on comorbidities and concomitant medications, the consistency of sICM levels across different subgroup analyses (age, sex, and clinical manifestations) supports the robustness of our findings. Another limitation is represented by the analysis of the sICM only, without studying the membrane‐bound counterparts. However, we have previously demonstrated that the soluble form of CTLA‐4 may be a good “proxy” for the activity of the membrane‐bound form, as showed by flow cytometry analysis.^50^

sICMs are significantly elevated in patients with PMR and GCA and effectively differentiate them from healthy controls, particularly PD‐1, PD‐L1, and PD‐L2. However, these molecules do not appear to differentiate patients with or without LVV, and they cannot be used to distinguish disease phenotypes. The increased serum concentrations of co‐inhibitory molecules during active disease may reflect an immune‐modulatory feedback mechanism aimed at controlling T cell hyperactivity, suggesting a complex involvement of adaptive immunity in addition to the innate immune response. Despite their consistent elevation across different patient subgroups (age, sex, and GC treatment status), their potential utility as biomarkers requires further validation. Larger prospective studies, including patients receiving specific treatments, are needed to determine clinically meaningful thresholds and assess their prognostic significance. These future studies may also provide deeper pathophysiologic insights into the mechanisms underlying PMR and GCA.

## AUTHOR CONTRIBUTIONS

All authors contributed to at least one of the following manuscript preparation roles: conceptualization AND/OR methodology, software, investigation, formal analysis, data curation, visualization, and validation AND drafting or reviewing/editing the final draft. As corresponding author, Dr Camellino confirms that all authors have provided the final approval of the version to be published, and takes responsibility for the affirmations regarding article submission (eg, not under consideration by another journal), the integrity of the data presented, and the statements regarding compliance with institutional review board/Declaration of Helsinki requirements.

## Supporting information


**Disclosure form**.

**Appendix S1:** Supplementary Information
