# Supplementary material for: Soluble Co‐Inhibitory Immune Checkpoint Molecules Are Increased in Patients With Polymyalgia Rheumatica Without Significant Correlations With Clinical Status: A Case‐Control Study
Source: ACR Open Rheumatol. 2025 May 9;7(5):e70045. doi: 10.1002/acr2.70045 (PMC12063068; doi:10.1002/acr2.70045)
Supplement: Supplementary file 2 — Appendix S1: Supplementary Information [file ACR2-7-e70045-s002.docx]

**Supplementary tables**

See the main tables and figures in the text for the used acronyms

| **Analyte** | **Absorbance Range (AU)** | **Concentration Range (ng/ml or pg/ml)** | **Intra-assay Precision** | **Inter-assay Precision** | **Deviation Between Duplicates** | **Detection Limit (ng/ml or pg/ml)** |
| --- | --- | --- | --- | --- | --- | --- |
| sCTLA-4 | 0.162 - 1.200 | 0.1 - 50 ng/ml | <8% | <6% | <10% | 0.1 ng/ml |
| sPD-1 | 0.162 - 3.000 | 0.156 - 10 ng/ml | <10% | 12% | <10% | 0.1 ng/ml |
| sPD-L1 | 0.162 - 2.800 | 0.156 - 10 ng/ml | <10% | 12% | <10% | 0.1 ng/ml |
| sPD-L2 | 0.162 - 2.600 | 0.062 - 10 ng/ml | <10% | 12% | <10% | 0.1 ng/ml |
| IL-6 | 0.162 - 1.400 | 6.1 - 500 pg/ml | <10% | <10% | <10% | 6.1 pg/ml |

**Supplementary Table 1**. Summary of Assay Performance, Precision, and Detection Limits for Serum Analytes

| **Diagnosis** | **PMR (n = 30)** | **PMR + GCA (n = 10)** | **p** |
| --- | --- | --- | --- |
| CTLA-4 | 4.2 (0.1-122.4) | 0.1 (0.1-21.8) | 0.12 |
| PD-1 | 1 (1-17.2) | 1 (1-12.5) | 0.31 |
| PD-L1 | 17.4 (2-411) | 28.9 (0.1-94.6) | 0.63 |
| PD-L2 | 3.2 (0.1-112.7) | 5 (0.1-105.7) | 0.65 |
| IL-6 | 94 (1-755.3) | 25 (1-226) | 0.08 |

**Supplementary Table 2.** Median concentrations and ranges of soluble immune checkpoints (sICMs) in patients with PMR versus patients with PMR and GCA.

| **Systemic manifestations (fever and/or weight loss)** | **present (17)** | **absent (23)** | **p** |
| --- | --- | --- | --- |
| CTLA-4 | 9.7 (0.1-122.5) | 0.1 (0.1-63.5) | 0.09 |
| PD-1 | 1 (1-17.2) | 1 (1-15.1) | 0.58 |
| PD-L1 | 17.5 (0.1-94.6) | 18.5 (2-411) | 0.74 |
| PD-L2 | 6.6 (0.1-105.7) | 3.2 (0.1-112.7) | 0.57 |
| IL-6 | 90 (1-755.3) | 60.9 (1-285.5) | 0.21 |

**Supplementary Table 3.** Comparison between serum concentrations of sICMs in patients with systemic manifestations (+) versus patients without systemic signs/symptoms (-).

| **Headache** | **present (8)** | **absent (32)** | **p** |
| --- | --- | --- | --- |
| CTLA-4 | 0.1 (0.1-21.8) | 1.8 (0.1-122.4) | 0.45 |
| PD-1 | 1 (1-1) | 1 (1-17.2) | 0.38 |
| PD-L1 | 33.5 (0.1-94.6) | 18 (2-411) | 0.76 |
| PD-L2 | 3.8 (0.1-105.7) | 3.2 (0.1-112.7) | 0.80 |
| IL-6 | 25 (1-226) | 76.8 (1-755.3) | 0.28 |

**Supplementary Table 4.** Comparison between serum concentrations of sICMs in patients with headache (+) versus patients without headache (-).

**Supplementary Table 5.** Comparison between serum concentrations of sICMs in patients with limb claudication (+) versus patients without limb claudication (-).

| **Limb claudication** | **present (3)** | **absent (37)** | **p** |
| --- | --- | --- | --- |
| CTLA-4 | 0.1 (0.1-21.8) | 1.3 (0.1-122.4) | 0.69 |
| PD-1 | 1 (1-1) | 1 (1-17.2) | 0.62 |
| PD-L1 | 36.5 (3-48.6) | 18.5 (0.1-411) | 0.74 |
| PD-L2 | 6.6 (3.5-11.3) | 3.2 (0.1-112.7) | 0.37 |
| IL-6 | 4.9 (1-22.9) | 87.1 (1-755.3) | 0.08 |

| **Peripheral arthritis** | **present (10)** | **absent (30)** | **p** |
| --- | --- | --- | --- |
| CTLA-4 | 15.8 (0.1-122.4) | 21. (0.1-63.5) | 0.31 |
| PD-1 | 1.0 (1.0-15.2) | 1 (1-17.2) | 0.54 |
| PD-L1 | 35.2 (2.0-94.6) | 17.4 (0.1-411.0) | 0.12 |
| PD-L2 | 6.6 (0.1-105.7) | 3.1 (0.1-112.7) | 0.42 |
| IL-6 | 93.2 (1.0-285.5) | 60.9 (1.0-755.3) | 0.82 |

**Supplementary Table 6**. Comparison between serum concentrations of sICMs in patients with arthritis (+) versus patients without arthritis (-).

| **Hand tenosynovitis** | **present (6)** | **absent (34)** | **p** |
| --- | --- | --- | --- |
| CTLA-4 | 9.7 (0.1-63.5) | 1.3 (0.1-122.4) | 0.24 |
| PD-1 | 1.0 (1.0-14.5) | 1 (1.0-17.2) | 0.68 |
| PD-L1 | 28.9 (3.0-101.6) | 17.5 (0.1-411.0) | 0.15 |
| PD-L2 | 5.0 (0.5-112.7) | 3.2 (0.1-105.7) | 0.37 |
| IL-6 | 45.0 (1.0-285.5) | 87.1 (1.0-755.3) | 0.58 |

**Supplementary Table 7.** Comparison between serum concentrations of sICMs in patienst with tenosynovitis (+) versus patients without tenosynovitis (-).

.

| **GC** | **GC-treated (6)** | **GC- naive patients (34)** | **p** |
| --- | --- | --- | --- |
| CTLA-4 | 10.6 (0.1-63.5) | 0.90 (0.1-122.4) | 0.32 |
| PD-1 | 1 (1-14.5) | 1 (1-17.2) | 0.54 |
| PD-L1 | 50.4 (5.2-101.6) | 18 (0.1-411) | 0.09 |
| PD-L2 | 9.2 (0.5-112.7) | 3.1 (0.1-105.7) | 0.10 |
| IL-6 | 45 (1-285.5) | 76.8 (1-755.3) | 0.75 |

**Supplementary Table 8.** Comparison between serum concentrations of soluble immune-checkpoints in GC-treated versus GC-naïve patients

| **Grade-3 LVV** | **Present (10)** | **Absent (30)** | **p** |
| --- | --- | --- | --- |
| CTLA-4 | 15.8 (0.1-122.4) | 10.2 (0.1-63.5) | 0.68 |
| PD-1 | 2.4 (1-15.2) | 2.8 (1-17.2) | 1 |
| PD-L1 | 28.9 (0.1-101.6) | 23.5 (0.1-112.7) | 0.31 |
| PD-L2 | 23.5 (0.1-112.7) | 14.2 (0.1-73.7) | 0.54 |
| IL-6 | 93.2 (1-226) | 141 (1-756) | 0.89 |

**Supplementary Table 9.** Comparison between serum concentrations of soluble immune-checkpoints in LVV+ versus LVV- patients.

| **Stratified analysis of AUC values** | | | |
| --- | --- | --- | --- |
| **Analysis by sex** | | |  |
| **Molecule** | **Males (n = 8)** | **Females (n = 32)** | **p-values** |
| CTLA-4 | 0.93 | 0.70 | 0.24 |
| PD-1 | 1.00 | 1.00 | 1.00 |
| PD-L1 | 1.00 | 0.96 | 0.25 |
| PD-L2 | 0.93 | 0.92 | 0.33 |
| IL-6 | 0.83 | 0.82 | 0.31 |
| **Analysis by age** | | |  |
| **Molecule** | **< 70 years ( n = 11)** | **≥ 70 years (n = 29)** |  |
| CTLA-4 | 0.75 | 0.74 | 0.83 |
| PD-1 | 1.00 | 0.99 | 0.92 |
| PD-L1 | 0.98 | 0.97 | 0.42 |
| PD-L2 | 1.00 | 0.89 | 0.18 |
| IL-6 | 0.70 | 0.85 | 0.28 |

**Supplementary Table 10.** Sensitivity analyses of soluble immune checkpoint molecules in PMR/GCA by sex and age subgroups.

| **Table of correlations** | **CTLA-4** | | **PD-1** | | **PD-L1** | | **PD-L2** | | **IL-6** | |
| --- | --- | --- | --- | --- | --- | --- | --- | --- | --- | --- |
|  | **rho** | **p** | **rho** | **p** | **rho** | **p** | **rho** | **p** | **rho** | **p** |
| age | 0.07 | 0.69 | 0.29 | 0.09 | -0.18 | 0.30 | 0 | 0.99 | 0.11 | 0.55 |
| TVS | -0.25 | 0.15 | 0.13 | 0.46 | 0.04 | 0.82 | 0.04 | 0.82 | -0.05 | 0.79 |
| TJS | 0.04 | 0.82 | 0.21 | 0.24 | **0.38** | **0.03** | 0.3 | 0.09 | -0.02 | 0.91 |
| disease duration | 0.29 | 0.09 | -0.06 | 0.76 | -0.18 | 0.31 | -0.15 | 0.4 | 0.2 | 0.25 |
| MS | -0.05 | 0.77 | 0.09 | 0.60 | -0.27 | 0.13 | -0.01 | 0.97 | -0.04 | 0.82 |
| CRP | -0.22 | 0.2 | -0.02 | 0.93 | -0.12 | 0.49 | 0.1 | 0.56 | 0.01 | 0.96 |
| ESR | 0.1 | 0.57 | -0.08 | 0.66 | -0.13 | 0.47 | 0.2 | 0.3 | 0.11 | 0.54 |
| PLT | 0.04 | 0.84 | -0.13 | 0.47 | -0.13 | 0.48 | 0.12 | 0.49 | 0.06 | 0.72 |
| Hb | -0.07 | 0.68 | -0.11 | 0.53 | 0.31 | 0.08 | 0.07 | 0.69 | -0.1 | 0.58 |
| WBC | 0.11 | 0.54 | -0.03 | 0.85 | -0.03 | 0.85 | 0.1 | 0.58 | -0.04 | 0.80 |

**Supplementary Table 11.** Correlations between serum immune checkpoint molecules with the quantitative clinical, laboratory and imaging features in the subgroup of GC-naïve patients (n = 34)

| **sICM stratified**  **according to diagnosis** | **PMR (n = 26)** | **PMR + GCA (n = 8)** | **p-value** |
| --- | --- | --- | --- |
| CTLA-4 | 4.2 (0.1-14.6) | 0.1 (0.1-3) | 0.12 |
| PD-1 | 1 (1-17.2) | 1 (1-12.5) | 1 |
| PD-L1 | 17.4 (5.8-28.1) | 20.5 (2.8-36.9) | 1 |
| PDL2 | 3.1 (1-9.6) | 3.1 (1.3-7.9) | 0.84 |
| IL-6 | 94 (21-7-189.6) | 25 (1-92.5) | 0.18 |

**Supplementary Table 12.** Median and interquartile ranges (IQR) of sICM in GC-naïve patients with isolated PMR and in patients with both PMR and GCA. Legend: IQR, interquartile range; sICM, soluble immune checkpoint molecules

| **Systemic signs and/or symptoms** | **Present (n = 16)** | **Absent (n = 18)** | **p-value** |
| --- | --- | --- | --- |
| CTLA-4 | 9.5 (0.1-20.8) | 0.1 (0.1-7.8) | 0.08 |
| PD-1 | 1.0 (1.0-1.0) | 1.0 (1.0-1.0) | 0.42 |
| PD-L1 | 17.4 (5.5-41.5) | 18.5 (5.8-25.6) | 0.56 |
| PDL2 | 5.3 (0.9-22.2) | 2.9 (1.1-3.6) | 0.43 |
| IL-6 | 126.5 (52.4-206.3) | 51.9 (2.8-150.7) | 0.13 |

**Supplementary Table 13.** Comparison between median concentrations with IQR of sICMs in individuals with systemic manifestations versus patients without systemic signs/symptoms (i.e. fever, weight loss) among GC-naïve patients.

| **Headache** | Present (n = 7) | Absent (n = 27) | p-value |
| --- | --- | --- | --- |
| CTLA-4 | 0.1 (0.1-5.8) | 2.3 (0.1-12.7) | 0.251 |
| PD-1 | 1.0 (1.0-1.0) | 1.0 (1.0-1.0) | 0.304 |
| PD-L1 | 30.5 (3.8-37.3) | 17.5 (5.9-27.5) | 0.798 |
| PDL2 | 3.5 (2.0-5.3) | 3.1 (1.0-9.8) | 0.815 |
| IL-6 | 27.0 (11.9-126.5) | 87.1 (11.8-188.4) | 0.440 |

**Supplementary Table 14.** Comparison between median concentrations with IQR of sICMs in individuals with headache versus patients without headache among GC-naïve patients.

| **Grade-3 LVV** | **Present (n = 10)** | **Absent (n = 24)** | **p-value** |
| --- | --- | --- | --- |
| CTLA-4 | 0.6 (0.1-9.3) | 3.4 (0.1-12.2) | 0.905 |
| PD-1 | 1.0 (1.0-1.0) | 1.0 (1.0-1.0) | 0.295 |
| PD-L1 | 13.9 (2.7-33.5) | 18.0 (5.9-28.7) | 0.545 |
| PDL2 | 2.5 (0.7-5.7) | 3.4 (1.2-9.8) | 0.461 |
| IL-6 | 64.7 (46.2-169.2) | 93.7 (1.0-176.6) | 0.581 |

**Supplementary Table 15.** Comparison between median concentrations with IQR of LVV+ versus LVV- patients.

| **Arthritis** | **Present (n = 10)** | **Absent (n = 24)** | **p-value** |
| --- | --- | --- | --- |
| CTLA-4 | 6.0 (0.1-13.3) | 0.4 (0.1-11.6) | 0.549 |
| PD-1 | 1.0 (1.0-1.0) | 1.0 (1.0-1.0) | 0.946 |
| PD-L1 | 15.7 (6.0-26.0) | 18.0 (4.2-32.0) | 0.955 |
| PDL2 | 2.3 (0.9-10.8) | 3.2 (1.2-7.2) | 0.664 |
| IL-6 | 76.8 (62.8-153.1) | 76.4 (1.0-188.4) | 0.790 |

**Supplementary Table 16.** Comparison between median concentrations with IQR of sICM between patients with arthritis versus patients without arthritis.

| **Tenosynovitis** | **Present (n = 6)** | **Absent (n = 24)** | **p-value** |
| --- | --- | --- | --- |
| CTLA-4 | 11.8 (4.2-15.5) | 0.1 (0.1-10.2) | 0.07 |
| PD-1 | 1.0 (1.0-1.0) | 1.0 (1.0-1.0) | 0.73 |
| PD-L1 | 18.3 (5.8-38.0) | 14.5 (4.2-22.9) | 0.74 |
| PDL2 | 1.8 (1.0-8.9) | 3.4 (1.3-9.2) | 0.62 |
| IL-6 | 63.3 (46.2-159.2) | 88.5 (6.5-188.4) | 0.98 |

**Supplementary Table 17.** Comparison between median concentrations with IQR of sICM between patients with tenosynovitis versus patients without tenosynovitis.

| Molecule | AUC | Median cut-off | | OR (median) | 75^th^ cut-off | OR (75^th^) | p-values |
| --- | --- | --- | --- | --- | --- | --- | --- |
| CTLA-4 | 0.73 | 0.10 | | 8.44 | 0.10 | 8.44 | 0.0003 |
| PD-1 | 1.00 | 0.10 | | NA | 0.10 | NA | < 0.0001 |
| PD-L1 | 0.97 | 0.35 | | 33.00 | 0.88 | 91.67 | < 0.0001 |
| PD-L2 | 0.93 | 0.10 | | 56.25 | 0.10 | 56.25 | < 0.0001 |
| IL-6 | 0.81 | 3.20 | | 3.25 | 4.65 | 9.03 | 0.02 |
| **PD-1 alternative analysis** | | | | | | | |
| Metric | | | Value | | | Interpretation | |
| Mean difference | | | 2.45 | | | Average elevation in cases vs controls | |
| Effect size | | | 0.78 | | | Large standardized difference | |
| Distribution | | | Non-overlapping | | | Complete separation between groups | |

**Supplementary Table 18.** Conditional logistic regression analysis in GC-naïve patients

Legend: OR, odds ratio from conditional logistic regression; median cut-off: control group median value used as threshold; 75^th^ cut-off: control group 75^th^ percentile value used as threshold; NA, not applicable due to complete separation; p-values, from chi-test square test for association


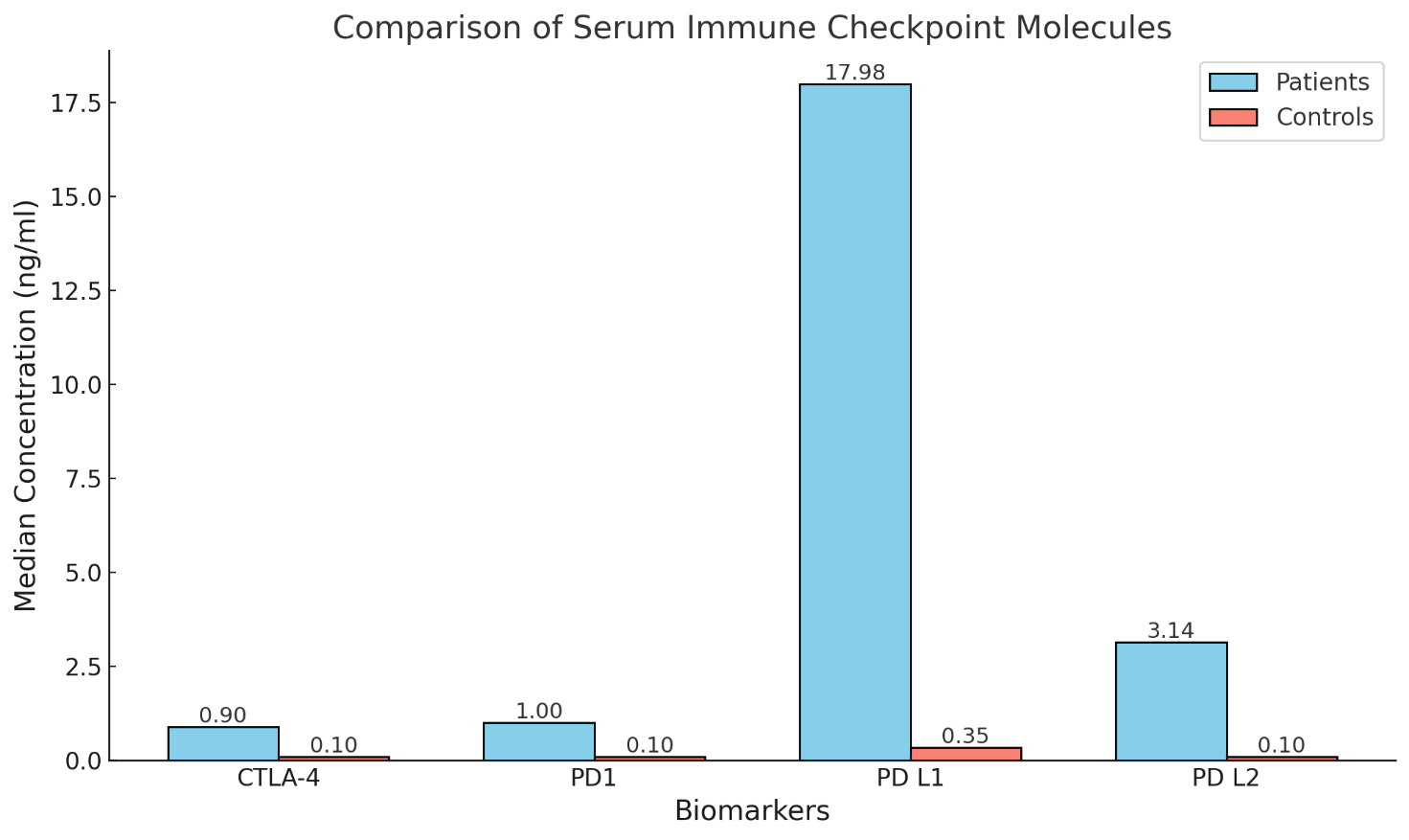


**Supplementary Figure 1.** Comparison of serum concentrations of sICMs between GC-naïve PMR/GCA patients (n=34) and age- and sex-matched healthy controls.
